# A Micronutrient Fortified Beverage Given at Different Dosing Frequencies Had Limited Impact on Anemia and Micronutrient Status in Filipino Schoolchildren

**DOI:** 10.3390/nu9091002

**Published:** 2017-09-12

**Authors:** Imelda Angeles-Agdeppa, Clarita R. Magsadia, Grant J. Aaron, Beate B. Lloyd, David C. Hilmers, Zulfiqar A. Bhutta

**Affiliations:** 1 Food and Nutrition Research Institute, Department of Science and Technology, 1632 Taguig, Philippines; clarita_magsadia@yahoo.com; 2 Formerly with the Global Alliance for Improved Nutrition (GAIN), Rue de Varembre 7, 1202 Geneve, Switzerland; grant.j.aaron@gmail.com; 3 Global Scientific and Regulatory Affairs, The Coca-Cola Company, Atlanta, GA 30301, USA; belloyd@coca-cola.com; 4 Baylor College of Medicine, Departments of Internal Medicine, Pediatrics, Global Initiatives, and Center for Space Medicine, Houston, TX 77030, USA; dhilmers@bcm.edu; 5 University of Toronto, Center for Global Child Health, The Hospital for Sick Children, Toronto, ON M6S 1S6, Canada; zulfiqar.bhutta@sickkids.ca

**Keywords:** hemoglobin 1, dose response 2, multi-nutrient fortified juice drink 3

## Abstract

This study evaluated the effects of a multi-micronutrient fortified juice drink given in different frequencies of consumption on hemoglobin (Hb) concentration of schoolchildren. Hb was measured in 2423 schoolchildren aged 6- to 9-years-old at baseline. All anemic children (*n* = 246) were randomly allocated into groups: Daily dose (HD: high dose), 5X/week (MD: Moderate Dose), 3X/week (LD: Low Dose) and unfortified (Control). Pre- and post-study measurements of micronutrients were collected from 228 children. At the endpoint, significant Hb increases were observed in all groups, but there was no significant difference between groups. There was a significant reduction in anemia prevalence in all groups from 100% to 36% (Control), 30% (LD), 23% (MD) and 26% (HD). No dose-response effect was observed in Hb in this population. Most likely, this resulted from better than expected micronutrient status and lower than expected severity of anemia and micronutrient deficiencies in this cohort. It is unlikely that the addition of a fortified beverage to school feeding programs in this population would have a positive impact. Whether such an intervention would be cost-effective as a preventative approach needs to be assessed. This study demonstrates the importance of targeting such interventions to appropriate populations.

## 1. Introduction

Nutritional anemia can be caused by different micronutrient deficiencies such as folate, riboflavin, vitamin A and cobalamin, but iron deficiency is the major contributor worldwide [1]. Iron deficiency is often caused by inadequate food intake and the presence of infections. In terms of the loss of healthy life expressed as disability-adjusted life years (DALYs), iron-deficiency anemia results in 25 million DALYs lost or 2.4% of the global total [2,3].

The prevalence of anemia in the Philippines is routinely monitored in national assessments [4]. Following the high prevalence of anemia observed in the 1998 assessments, the Philippine government passed the Food Fortification Law in 2000. The law requires mandatory fortification of staples like rice (with iron) and oil (with vitamin A) while voluntary fortification with iron, vitamin A or iodine was encouraged for processed foods. The National Food Authority (NFA) for rice, the Food and Drug Admisitration (FDA) for wheat flour, and the Philippine Coconut Authority (PCA) for cooking oil are the government agencies that are responsible for monitoring the implementation of fortification of staple foods. For processed products, the Department of Health (DOH) puts a seal of excellence (*Sangkap Pinoy Seal*) on food labels if the fortification level has reached 30% of the recommended requirement of the major micronutrients such as iron, vitamin A and iodine. The FDA is the overall monitoring agency for product registration. From the period of 2003 to 2008, the prevalence of anemia in children aged 6 to 12 years old decreased from 29% to 20% [4]. Despite this, anemia and iron deficiency remain problematic in certain population groups: 6 to 11 months (56%), pregnant women (42%), lactating women (31%), ≥60 years (32%). In the same 2008 nationwide Food Consumption Survey, only 8.8% of the households surveyed met the Estimated Average Requirement (EAR) for iron [4]. The current study was developed in 2013, the period when the National Survey was in progress; hence, prevalence data from 2008 was used as a reference.

Improved diets, affordable food fortification and supplementation programs integrated into carefully planned community, school and health initiatives directly improve nutrition and ameliorate the human and economic consequences of an undernourished population at its core [5]. Among food intervention programs, food fortification is highlighted as one of the most cost-effective health solutions to fight malnutrition among children and iron deficiency in women [6,7,8]. Our aim was to assess the addition of a multi-micronutrient fortified beverage to school feeding programs in order to inform cost-effective scale-up. We set out to evaluate the dose response effect of a multi-micronutrient, fortified juice drink at different daily intervals of consumption on Hb concentration and biomarkers of micronutrient status such as ferritin, retinol binding protein (RBP), zinc, and vitamins C and D of schoolchildren aged six to nine years old after 120 feeding days.

To our knowledge, there are no studies that have assessed the lowest frequency of fortified juice drink consumption that has a significant effect on Hb concentration and micronutrient status. Understanding the optimal dosing and frequency is essential for ensuring a meaningful intervention under real-world conditions where costs need to be minimized and compliance varies.

## 2. Materials and Methods

### 2.1. Study Design and Sampling Size

The study was conducted in 6 public elementary schools in 2 municipalities of Rizal province in the Philippines. The schools were selected because they are adjacent to each other and have similar socio-economic and environmental characteristics.

**Figure 1:**
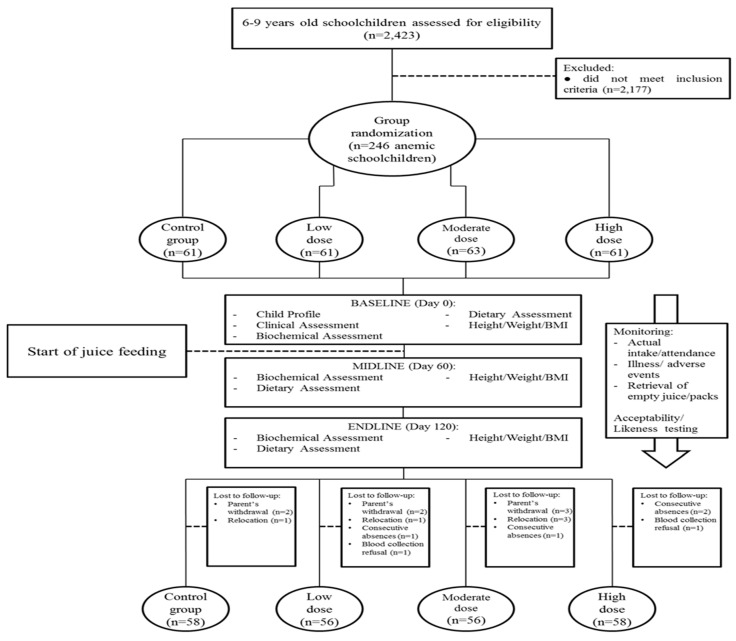
Consort flow diagram.

All eligible children aged 6 to 9 years old were invited to participate in the study; however, only children with written parental consent were selected for screening. Children not suffering from any infections (diarrhea, fever, acute respiratory infections) or severe acute malnutrition during the time of examination and for the two weeks prior to the assessment period were further assessed for Hb concentration. Children found to be anemic with Hb > 70 g/L to < 120 g/L (altitude adjusted norm) [9] and with normal weight-for-age *z*-score (WAZ) less than −2 to +2 standard deviation [10] were included as participants in this study. Children with acute illnesses, severely underweight (WAZ ≤ 3 SD), profound anemia (Hb < 70 g/L), history of a blood disorder or malaria, currently taking vitamins and not willing to stop during the intervention, and participants in a nutrition program during the last 4 months were excluded, and children with acute illnesses or severe anemia were referred to the nearest health facility for management (Figure 1). Thirty-five children per group were needed for this study. The sample size was calculated to detect a minimum change of 7 g/L in Hb concentration with an estimated SD of 9 g/L, a confidence interval of 95%, and a power of 90% to allow for a 30% attrition rate, the sample size was increased to 50 per group [11,12,13].

A total of 2423 eligible children were screened for anemia prior to the study. Those with anemia were randomly allocated into four groups in a double-masked, placebo-controlled manner. Each child received one color-coded box containing seven pouches of 200 mL unbranded foil pouch with the same color code per pack. The high dose (HD) group received fortified juice drink 7 days a week; the Moderate Dose (MD) group received 5 fortified and 2 unfortified juice drinks; the Low Dose (LD) group received 3 fortified and 4 unfortified juice drinks; and the Control group received only unfortified juice drink. Since 7 similar color coded pouches were contained in one box as the weekly allocation for each child, the researchers were blinded as to the day the unfortified juice was given in the LD and MD groups. Therefore, the exact spacing in which the fortified drink was consumed was unknown.

The study product is a multi-micronutrient fortified juice drink, enriched with iron (in the form of ferrous gluconate), zinc, vitamin A, C, D and B-complex. (Table 1) An independent Expert Committee designed the study formulation, and nutrient analysis was verified by an external laboratory. A responsible person, not a member of the research team, directly supervised the packing and ensured that the correct number of pouches of fortified and/or unfortified juice drinks were in the box. The codes for each group were safeguarded until the end of the study. The juice drink was directly administered during the morning school break between 9:00 and 10:00 a.m., leaving about 2 h before the child’s next food consumption as the children go home for lunch at 11:30 a.m. or 12:00 p.m. The juice drink was given with a locally-manufactured, non-whole grain, butter or cream-filled 30 g biscuit with less than 30 kcal. The juice drink and biscuits were given five days per week under a supervised regimen for 120 days (Figure 1).

### 2.2. Monitoring of Intake

The children were required to consume the entire serving throughout the duration of the study. If classes were suspended or the child was absent for a maximum of 3 successive days, juice allocation was resumed when the child returned to class. This child was then given 2 juice drinks and 2 biscuits per day taken early in the morning before his class starts and another pack was given before the child goes home until all the missing doses were consumed. If children were absent for more than three days and were counted as absent by the class teacher due to illness, a home visit was conducted to deliver the allocation. However, if the residence was far from the school, the field teams asked the parents to claim the child’s allocation at the school and were instructed on how to administer the beverage. Continuous absence for two weeks constituted a drop-out and the child was dis-enrolled. The cause of absence or drop out, such as illness, was recorded in the Individual Case Report Form (ICRF).

### 2.3. Measurements

A Manual of Operations and Instructions was developed to maintain standards during data collection, implementation, data organization and management. Face-to-face interviews were done by trained field teams to collect information on household socio-economic and demographic data and food intake. Household socio-economic and demographic data were obtained only at baseline while food intake was calculated at baseline, midpoint and endpoint by trained nutritionists using the 24-h food recall method on two non-consecutive days (weekday and weekend). The presence of the child during the interview helped to validate food intake outside the home [15]. Food intakes were translated into energy and nutrient values using the Individual Dietary Evaluation System (IDES) developed by the Department of Science and Technology–Food and Nutrition Research Institute (DOST–FNRI). Adequacy of intakes was computed as the percentage of actual nutrient intake to values published in the Recommended Nutrient Intake (RNI) of the PDRI [14].

Non-fasted blood samples were drawn in the morning between 7:00 a.m. and 11:30 a.m. Phlebotomy was done by trained medical technologists from DOST–FNRI in a clean room in the school facility to avoid environmental contamination [16]. Venous blood samples were collected using disposable 22–24 gauge syringes and transferred to trace element-free BD (Becton Dickinson) Vacutainer^®^ (BD Company, Franklin Lakes, NJ, USA) PLUS Blood Collection Tubes. Approximately 5 mL of blood was collected from each child to measure hemoglobin concentration, serum transferrin receptor, serum ferritin, retinol binding protein (RBP), zinc and vitamin C and D levels. At the FNRI ISO Laboratory, Taguig City, Philippines, serum zinc was analyzed using a Flame Atomic Absorption spectrometer. The recommended cut-off for children below 10 years old is 9.9 μmol/L (65 μg/dL) [17]. Vitamin C was measured spectrophotometrically at 520 nm. Serum vitamin C concentration (mg AA/dL) was computed from the regression of absorbance vs. concentration of the standard [18]. In this study, a cut-off value of <0.20 μg/dL defines vitamin C deficiency [18]. Vitamin D was analyzed using electrochemiluminescence immunoassay (ECLIA) for quantification of serum 25 hydroxy vitamin D. In this study, deficiency is described as <75 nmol/L [19].

Serum samples were collected in a polyethylene micro-centrifuge tube separately for each analysis. All samples were transported in a cool box with wet ice to the FNRI Laboratory where these were stored immediately at −40 °C until analysis. All biochemical markers were analyzed at baseline and the end of the study except for hemoglobin, in which midpoint assessment was also accomplished.

Hemoglobin concentration was measured for the 2423 children who were potential subjects. Those who were anemic were invited to participate in the intervention study. The Hb concentration at screening served as the baseline Hb. All Hb measurements (baseline, midpoint and endpoint) were performed in an ISO (International Standards Organization) accredited FNRI Biochemical laboratory using the cyanmethemoglobin method and accuracy was determined by measuring the hemoglobin concentration of control blood [20,21]. In this study, hemoglobin cut-off points for anemia at sea level were used; hence, a hemoglobin concentration of >70 g/L to <120 g/L [9] was considered anemic.

Remaining blood samples were centrifuged within 1–2 h after phlebotomy at 2500 to 3000 rpm for 15–20 min. Serum ferritin, serum transferrin receptor RBP, C-reactive protein (CRP) and alpha-1-acid glycoprotein (AGP) samples were packed under maximum quality control conditions for analysis using the Sandwich ELISA technique [22] at VitMin Laboratory in City, Germany. Serum ferritin < 30 µg/L indicated iron deficiency [9]; serum transferrin receptors > 8.3 mg/L demonstrated low iron storage [23]. CRP values > 10 mg/L and AGP > 1 g/L [24] described the presence of acute and chronic infections, respectively. Using CRP and AGP, an adjustment to the ferritin values was made utilizing Thurnham’s correction factor (CF) [23].

### 2.4. Ethical Considerations

The study was approved by the Food and Nutrition Research Institute Institutional Ethics Review Committee and was carried out in accordance with the Declaration of Helsinki, guided by the Council for International Organizations of Medical Sciences Ethical Guidelines for Biomedical Research Involving Human Subjects [25] and the National Guidelines for Biomedical/Behavioral Research [26]. The parents were clearly informed of the objectives, procedures, risks and benefit that their children may encounter by participation in the study. Individual signed parental Informed Consent Forms (ICF) was obtained.

Any adverse events (AE) and/or complaints experienced by the children based on self-report were recorded in a prescribed AE Form. As per protocol, any reported AE, such as abdominal pain, diarrhea or gastric irritation, was to be referred to the Study Physician. However, during the course of the study, no serious adverse events were reported.

### 2.5. Statistical Analysis

Pearson’s χ^2^ test was performed to determine if the demographic variables were associated with groups. For socio-economic characteristics, *f*-test using one-way analysis of variance (ANOVA) was done to determine the differences among groups; Pearson’s χ^2^ test was used to determine the association between the socio-economic variable and groups.

For dietary assessment, means and standard errors were computed. An *f*-test using one-way ANOVA was used to test intergroup changes while a *t*-test using paired samples was done to test the intragroup changes. The variations in proportion of children meeting the RENI from baseline to endpoint were calculated for each nutrient and were tested using Pearson’s χ^2^ test. To determine if the proportion of subjects not meeting the RNI of a micronutrient from baseline to endpoint remained significant, McNemar’s test was used.

The means and standard error of nutritional biochemical markers were computed. First, we tested the normality of the data. *f*-test using one-way ANOVA was used to test the differences among groups while *t*-test using paired samples was done to test the differences between time periods. To test the difference within groups, we subtracted the endpoint from the baseline values and tested the mean intragroup difference by time periods using one-way ANOVA. An intragroup difference was done to determine the increase or decrease in individual biomarkers at different time periods when significant group-wise comparisons were observed. The association between the prevalence of deficiency and groups was determined using Pearson’s χ^2^ test. To determine if the proportion of inadequate levels of biochemical markers from baseline to endpoint remained significant, McNemar’s test was used. Adjustments were made for serum ferritin [27] and to account for the effect of inflammation.

## 3. Results

A total of 246 or 10% of 2423 aged 6- to 9-year-old children were anemic and participated in this study (Figure 1). Only 228 (92.7%) had a complete data set available for analysis from baseline to endpoint. The total drop-out rate was similar across groups and was within the assumed 30% attrition rate [15,16]. The reasons and number of children who dropped-out were: withdrawal by parents because of inability to come for feeding during holidays (*n* = 7); relocation to another area (*n* = 5); absence for 2 consecutive weeks (*n* = 4); and refusal for endpoint phlebotomy (*n* = 2). The mean hemoglobin at baseline, age and sex of drop-outs were not statistically different from the remaining subjects.

The mean age of the children was 7.32 years old, and the age and gender distributions were similar between groups. The mean monthly family income was approximately Php 14,000.00 ($300 U.S.) with mean daily food expenditure of Php 260.00 ($5.50 US) with an average household size of six persons. No significant differences between groups were found in mean monthly family income (*p* = 0.266), daily food expenditure (*p* = 0.201), mean age of the respondents (*p* = 0.325) and household size (*p* = 0.547). The percentage of families with > 3 earning family members was significantly higher in HD than in the Control (*p* < 0.05). The percentage of families that owned residential lots was higher in the Control group than in the HD; thus, the percentage of renting the residential lot is higher in HD than in the Control group (Table 2).

At baseline, hemoglobin concentration of children ranged from 10.0 to 11.4 g/dL. Mean hemoglobin concentration was significantly lower in the control group (10.8 g/dL) than in the fortified groups (11.1 g/dL) at baseline. The clinical relevance of this difference is marginal in terms of anemia severity (i.e., all groups fell in the same category of mildly anemic). At endpoint, mean hemoglobin had significantly increased in all groups with the highest increase in HD of 1.0 g/dL. The prevalence of anemia, which was 100% at baseline by study design, significantly decreased in all groups with MD having the highest reduction (76.8%) followed by HD (74.1%) while the Control group had 64% and the LD 70%, but no significant intergroup changes were observed (Table 3).

Mean adjusted serum ferritin of the fortified groups increased from baseline to endpoint ranging from 2.2 µg/L both in LD and HD to 4.5 µg/L in MD, while Control increased by 3 µg/L. However, there were no significant differences between groups (*p* > 0.05). Serum transferrin receptor levels did not differ significantly among all groups at baseline and endpoint. Using both serum transferrin receptor (sTrf > 8.3 mg/L) and corrected serum ferritin (SF < 30 µg/L) as indicators of iron deficiency, only two children met this criteria (Control = 1; MD = 1) at baseline. Using adjusted serum ferritin level (SF < 30 µg/L) alone as the criterion, 16 children were iron deficient at baseline but utilizing SF < 15 as the cut-off, only 3 children were found to be iron deficient. At endpoint, only one child (from the MD group) remained iron deficient. Changes in the prevalence of iron deficiency as measured by serum ferritin, serum transferrin, and body iron stores, from baseline to endpoint were not significant between and within groups during the study. In addition, the prevalence of anemia and iron deficiency was not associated with the frequency of administration, which indicates that no dose response effect was observed (Table 3). For other biomarkers, results showed that from baseline to endpoint, there was a significant increase in retinol binding protein (RBP) in all groups; vitamin D status improved in all except LD; vitamin C only improved in those groups consuming the fortified beverage; zinc status was only improved in MD (Table 4).

Dietary intakes at baseline showed that only the mean of the two-day intake of niacin was significantly different between groups with the control group having the lowest (10.2 mg), while HD had the highest intake (14.6 mg). Mean iron intake at baseline was similar in all groups. At endpoint, the mean iron intake in the control group remained similar to the baseline value (from 7 to 7.6 mg) while the fortified groups had significantly increased intakes with the HD having the highest increment (10.12 mg) of which 42% came from the juice drink. In other groups, LD increased by 8.95 mg and MD by 9.05 mg with about 23.1 mg and 35.4 mg contributed by the juice drink, respectively. Vitamin C intake increased significantly in all the fortified groups but not in the Control group. All groups consuming the fortified juice drink had a significantly increased proportion of children meeting the Estimated Average Requirement (EAR) for iron, vitamin C, thiamin, niacin and riboflavin as compared with baseline. The study feedings with iron contributed to 23% (LD), 35% (MD) and 42% (HD) of the intake for total iron intake, respectively. The fortified beverage was considered an important contributor to intake of essential micronutrients often lacking in the diet, including iron, thiamin, riboflavin, vitamin C, folate, potassium calcium and vitamin D. All groups consuming the fortified juices had a significantly increased proportion of children meeting the Estimated Average Requirement (EAR) for iron, vitamin C, thiamine, niacin and riboflavin from baseline. (Table 5) The total dietary intake could not be estimated for the other nutrients, as these data were not available per the existing food composition databases. Results on awareness of fortified food products or the Sangkap Pinoy Seal (SPS) showed that, among groups, more than half of the guardians (68%) were not aware of these products. The MD group had the highest percentage (69.6%). Among those who were aware, the HD group had the lowest percentage of guardians (80.0%) serving fortified foods to children. However, test for comparisons did not show a significant difference between groups. More than half of the guardians from the Control group (65%) stated that they serve these products because they “make my child healthy” while only 37.5% from the HD group did so. The source of fortified products was mainly from the grocery stores, but the control and LD groups also buy from public markets and village variety stores.

## 4. Discussion

Of the total 2423 children screened, only 10% (246) were anemic. This finding was surprisingly low given the estimated prevalence of anemia from the 2008 National survey. There were 228 (92.7%) children from whom a complete data set was collected from baseline to endpoint. The total dropout rate was similar across groups and was within the 8% assumed attrition rate. The characteristics of drop-outs including age, sex, and basal hemoglobin did not change the pooled values of the remaining subjects within their respective groups.

The present study showed no significant hemoglobin differences in between group comparisons. Hemoglobin concentrations significantly increased in all groups including the Control group at endpoint (*p* < 0.001), but HD had the highest absolute mean increase in hemoglobin (10 g/L). Consequently, the percentage decline in the prevalence of anemia was similar in all groups 64% (Control), 70% (LD) 77% (MD) and 74% (HD). In addition, no significant difference between groups comparison in serum ferritin and serum transferrin receptors was observed at endpoint. For other biomarkers, from baseline to endpoint, there was a significant increase in retinol binding protein (RBP) in all groups; vitamin D status improved in all except LD; vitamin C only improved in those groups consuming the fortified beverage; zinc status was only improved in MD. Two relevant questions arise from these results: why was there an improvement in the prevalence of anemia in all groups, and why did we not see a dose-response effect from the intervention?

The severity of anemia at baseline was mild in all groups. It is possible that this could be a seasonal trend. The baseline data was collected in July, a month after the one and a half month school break. Children might have increased their iron intake due to better maternal care.

The comparable significant increments in Hb in all groups are similar to earlier published intervention studies with fortified foods. In a trial in India, despite receiving no micronutrient premix, the control group exhibited improvements in hemoglobin (121.7 to 122.5 g/L), and reduction in iron-deficiency anemia (9.8% to 4.3%), which was only marginally different than the intervention group [28]. Similar results were also observed in Indonesia [29], in Vietnam [30], and in Brazil [27]. The authors postulated that communicating the goals of the study to the participants’ parents or caregivers could have caused an intervention bias that led them to modify and improve their child’s dietary intake. We could also attribute some of the improvement of Hb in the control group to what may be called “study effect”, wherein the intervention itself changed the behaviors of parents to the benefit of their children’s nutritional status. In addition, parents in the Control group had slightly better knowledge about heath practices and nutritional fortification and had better access to fortified products. This group had the highest percentage with a single earning family member, usually the father. Hence, mothers were left behind to care for the children as compared with the intervention groups and presumably were more attentive to the dietary needs of their child during the study. In the HD, a higher percentage of families had two to three members working, including the mother, but, despite this, the family income was similar to the Control group. In general, the socio-economic status of the Control group was better than the intervention groups. Socio-economic variables may determine purchasing power, food choices, and time available to mothers to care for their children’s needs at home. These advantages might have been further accentuated during the course of the study.

The lack of dose response could be attributed to several possibilities that would account for this finding. First, the subjects were rather iron-replete as only nine or fewer subjects had actual iron deficiency at baseline in each group. This may be due to previous exposure to iron fortified foods. It would not be expected that the response to a fortified drink would be as robust as it would in a more iron deficient cohort; Second, the dose of iron in the beverage was relatively low and, although beneficial as a preventative measure for the progression to iron deficiency, may not have been high enough to have a statistically significant effect in this study population. The duration of the study was relatively short and might have required a longer period of administration to demonstrate a positive benefit. Prior studies have shown that iron status does not change rapidly with fortification [31], especially when the level of the added nutrient is low. A bioavailability study using stable isotopes in Peruvian children aged 6–9 years old children with a multiple micronutrient fortified beverage containing an iron dose of 7 mg/serving revealed that iron absorption was significantly lower with a meal than without (9.8 ± 6.7% versus 11.6 ± 6.9%, *p* = 0.04) [32]. In our study, iron content per 200 mL serving was lower and only provided 50% (5.4 mg) of the intake in the HD, 38% or 3.9 mg (MD) and 23% or 2 mg (LD). It could also be possible that the biscuit that was consumed at the same time as the beverage had a mild effect on iron absorption.

Other considerations that may contribute to the findings include the interactions of micronutrients in an aqueous solution. In aqueous solutions and at higher intake levels, competition between micronutrients with similar chemical characteristics and uptake by non-regulated processes can take place. These interactions have clearly been demonstrated in experimental absorption studies and to some extent have been confirmed in supplementation studies [33]. Negative effects of zinc supplementation on iron and copper status have been reported [34] although at higher doses than used in our study. Studies on human subjects have shown that calcium (Ca) can inhibit iron (Fe) absorption, regardless of whether it is given as Ca salts or in dairy products. However, a thorough review of studies on humans in which Ca intake was substantially increased for long periods shows no changes in hematological measures or indicators of iron status. Thus, the inhibitory effect may be of short duration and there also may be compensatory mechanisms [35]. The juice drink was fortified with calcium with contributions ranging from 66 mg in the LD, 110 mg in MD to 154 mg in the HD. With the limited data that we have from our study, it is not possible to ascertain whether this interaction is only of theoretical interest and not of clinical significance.

The role of hepcidin and its regulation of iron absorption may also play a role in the optimum dosing frequency of the beverage. In a study using stable isotopes of iron, showed that iron supplements at doses of 60 mg Fe as FeSO_4_ in young women induced higher hepcidin levels in the blood for up to 24 h and were associated with lower iron absorption on the following day. This raises the possibility that optimal dosing may not be on a daily basis but with higher doses on alternate days. While the amount of iron contained in the beverage was much lower than was used in the study by Moretti, it is possible that there is a subtle effect mediated by hepcidin that might have played a role in our results [36]. The influence of hepcidin on iron absorption and optimal dosing of iron-fortified beverages remains to be investigated. Finally, concomitant deficiencies such as folic acid, and the prevalence of hemoglobinopathies in the study population may have minimized the effect of the intervention. In the 2013 Nationwide Nutrition Survey, a subsample thalassemia study in Metro Manila, Philippines revealed that the prevalence of hemoglobinopathies among anemic individuals was 27.8% and alpha thalassemia was 20.8% [37].

### 4.1. Limitations of This Study

The juice drink is fortified with folic acid, and B-vitamins, but no laboratory analysis of B12 levels, serum or red cell folate was done; hence, no further conclusions can be made about micronutrient deficiencies that could have contributed to anemia among the study children. In fact, the current Philippine Food Composition Table (FCT) does not include values for folate and other nutrients in the juice drink; therefore, the authors could not analyze the contribution of these nutrients to total intake. The prevalence of hemoglobinopathies in the study population was also not investigated. Various forms of thalassemia, for example, could have resulted in mild anemia, yet would not have responded to iron supplementation.

### 4.2. Further Work

Based on the lower than expected prevalence of anemia and micronutrient deficiencies in the Philippines 2013 national survey and in this study population, and the availability of other food-based interventions, a decision should be made on whether it is necessary to use fortified beverages in this setting. Intervening (or not) based on population needs is critically important both in terms of impact and ethics. The results from the 2013 national assessment showed a decrease of 8.4% in the prevalence of anemia from the previous 2008 survey. This information was not available during the time of the study; however, in hindsight, it does appear that existing strategies to reduce anemia and micronutrient deficiencies in the population are effective. Further work should assess whether more targeted approaches may be appropriate among those not reached, and/or whether other population groups besides children (women of child-bearing age, for example) have not benefited from existing micronutrient strategies.

This study attempted to address the question of micronutrient dose and frequency in a fortified beverage; however, the results leave this question unanswered. Further work to optimize dose and frequency of fortification interventions targeted to children (and other population groups) is important to inform cost-effective scale-up strategies in real world settings.

Although the study found minimal impact in the study population, it is still likely that the intervention may have a preventative effect. Further work to assess the appropriateness of the current intervention as a preventative measure is warranted. From a cost-effectiveness perspective, prevention of anemia and micronutrient deficiencies is favored over treatment approaches.

## 5. Conclusions

There is good evidence to suggest that when micronutrient deficiencies are present, micronutrient interventions, including micronutrient-fortified beverages, can have a positive impact [17]. The current study found minimal benefit from the intervention across study groups. The most plausible explanation for these findings is the better than expected micronutrient status and the lower than expected prevalence and severity of anemia and micronutrient deficiencies in the study population. The dose of iron in the beverage is relatively low and, although beneficial as a preventive measure for progression to iron deficiency, probably is not high enough to have a statistically significant effect in this relatively iron-replete population. The duration of the study was short and may have required a longer period of administration to demonstrate a positive benefit. Other considerations that may contribute to the findings include the interactions of micronutrients in an aqueous solution, the role of hepcidin in inhibiting iron absorption after dosing, concomitant deficiencies such as folic acid, and the prevalence of hemoglobinopathies in the study population. Given the results of this study, and the lower prevalence of anemia and micronutrient deficiencies found in the 2013 national survey, it is unlikely that the addition of a fortified beverage to school feeding programs would carry a significant positive impact. The question of whether such an intervention would be cost-effective as a preventative approach needs to be assessed. As demonstrated by the current study, it is critically important to intervene only when there is a justified need in the population.

## Figures and Tables

**Table 1:** Nutrient composition ^a^ of the unfortified and fortified drinks.

Nutrients	Unfortified		Fortified	
	Amount/200 mL Serving	% RENI ^b^ for 6–9 Years	Amount/200 mL Serving	%RENI ^b^ for 6–9 Years
Energy (kcal)	63.43	3.96	60.34	3.77
Carbohydrates (g)	15.74	99.23	15.04	99.70
Vitamins				
*Beta Carotene (µg RE)*	23.78	5.94	97.00	24.25
*Thiamin (mg)*	0.01	1.43	0.41	58.57
*Riboflavin (mg)*	0.00	0.29	0.34	48.57
*Niacin (mg)*	0.03	0.29	6.90	76.67
*Vitamin B6 (mg)*	0.00	0.00	0.86	114.23
*Dietary Folate (µg)*	0.00	0.00	165.00	55.00
*Vitamin C (mg)*	3.13	34.73	94.90	210.89
*Vitamin D (IU)*	0.00	0.00	145.90	72.95
*Vitamin E (IU)*	0.00	0.00	4.20	31.82
Minerals				
*Calcium (mg)*	0.00	0.00	154.00	22.00
*Iron (mg)*	0.02	0.16	5.43	54.30
*Zinc (mg)*	0.00	0.00	5.60	109.80
*Phosphorus (mg)*	4.03	0.81	64.40	12.88
*Magnesium (mg)*	0.79	0.88	0.79	0.88
*Potassium (mg)*	33.10	2.07	33.00	2.06

^a^ Based on the results of analysis; ^b^ Based on the RENI (Recommended Energy and Nutrient Intake) of the PDRI (Philippine Dietary Recommended Intake) [14].

**Table 2:** Distribution of children by socio-economic and demographic characteristics at baseline by group.

Characteristics	Control	Low Dose (LD)	Moderate Dose (MD)	High Dose (HD)	Total	*p*-Value ^1^
	(*n* = 58)	(*n* = 56)	(*n* = 56)	(*n* = 58)	(*n* = 228)	
	*n* (%)	*n* (%)	*n* (%)	*n* (%)	*n* (%)	
Age of study child, mean (SE)	7.3 (0.1)	7.2 (0.1)	7.4 (0.1)	7.3 (0.1)	7.3 (0.0)	0.90
Age Group						0.60
*6*	24 (41.4)	25 (44.6)	26 (46.4)	23 (39.7)	98 (43.0)	
*7*	20 (34.5)	16 (28.6)	16 (28.6)	23 (39.7)	75 (32.9)	
*8–9*	14 (24.1)	15 (26.8)	14 (25.0)	12 (20.7)	55 (24.1)	
Sex						0.60
*Male*	29 (50.0)	24 (42.9)	23 (41.1)	30 (51.7)	106 (46.5)	
*Female*	29 (50.0)	32 (57.1)	33 (58.9)	28 (48.3)	122 (53.5)	
Age of Respondent, mean (SE)	36.6 (1.3)	34.5 (1.4)	37.5 (1.1)	35.6 (1.1)	36.0 (0.6)	0.25
Family Type						0.92
*Nuclear*	39 (67.2)	38 (67.9)	40 (71.4)	38 (65.5)	155 (68.0)	
*Extended*	19 (32.8)	18 (32.1)	16 (28.6)	20 (34.5)	73 (32.0)	
Household Size, mean (SE)	6.3 (0.3)	6.5 (0.4)	5.9 (0.3)	6.4 (0.2)	6.3 (0.2)	0.55
Main Occupation of Father						0.15
*Production-related*	17 (29.3)	10 (17.9)	5 (8.9)	7 (12.1)	39 (17.1)	
*Service-related*	26 (44.8)	20 (35.7)	25 (44.6)	24 (41.4)	95 (41.7)	
*Transportation-related*	6 (10.3)	9 (16.1)	11 (19.6)	11 (19)	37 (16.2)	
*Clerical, business, technical-related*	9 (15.5)	17 (30.4)	15 (26.8)	16 (27.5)	57 (25.1)	
Number of Earning Family Members						0.02 *
*1 Member*	24 (41.4)	29 (51.8)	31 (55.4)	34 (58.6)	118 (51.8)	
*2 Members*	31 (53.4)	21 (37.5)	24 (42.9)	14 (24.1)	90 (39.5)	
*3 or More Members*	3 (5.1)	6 (10.7)	1 (1.8)	10 (17.1)	20 (8.8)	
Estimated Total Monthly Family Income in Philippine Peso, mean (SE)	12,503.62 (1,088.12)	13,164.79 (1,020.72)	15,146.43 (1,271.31)	14,944.62 (1,152.04)	13,936.08 (569.42)	0.27
Average Daily Food Expenditure in Philippine Peso, mean (SE)	242.86 (13.56)	276.64 (21.10)	235.54 (14.17)	269.66 (14.30)	256.18 (8.04)	0.20
Status of residential lot						0.02 *
*Owned*	26 (44.8)	21 (37.5)	18 (32.1)	14 (24.1)	79 (34.6)	
*Renting*	19 (32.8)	12 (21.4)	9 (16.1)	20 (34.5)	60 (26.3)	
*Living with Relatives (Free)*	12 (20.7)	17 (30.4)	17 (30.4)	14 (24.1)	60 (26.3)	

^1^ Pearson’s χ^2^ test of independence and *f*-test; * *p* < 0.05.

**Table 3:** Mean values and percent distribution of iron biomarkers of schoolchildren by group by period.

Biomarkers	Control (*n* = 58)	Low Dose (*n* = 56)	Moderate Dose (*n* = 56)	High Dose (*n* = 58)	*p*-Value ^1^
	Mean (SE)	Mean (SE)	Mean (SE)	Mean (SE)	
Hemoglobin (g/dL)					
*Baseline*	10.8 (0.1)	11.1 (0.1)	11.1 (0.0)	11.0 (0.1)	0.01 *
*Endpoint*	11.7 (0.1)	11.9 (0.1)	11.9 (0.1)	12.0 (0.1)	0.50
*Difference*	0.9 (0.1)	0.9 (0.1)	0.8 (0.1)	1.0 (0.1)	0.47
No. (%) Anemia					
*Baseline*	58 (100)	56 (100)	56 (100)	58 (100)	1.00
*Endpoint*	21 (36.2)	17 (30.4)	13 (23.2)	15 (25.9)	0.44
*Difference*	37 (63.8)	39 (69.6)	43 (76.8)	43 (74.1)	
Serum ferritin(μg/L)					
*Undajusted for inflammation Baseline*	66.4 (5.1)	68.7 (4.4)	73.5 (5.6)	68.4 (3.8)	0.75
*Endpoint*	65.5 (4.3)	72.0 (4.3)	74.2 (4.8)	71.4 (3.3)	0.50
*Difference*	0.9 (4.5)	3.2 (3.5)	0.7 (4.6)	2.9 (3.8)	0.88
*Adjusted for inflammation ^a^*					
*Baseline*	59.0 (4.1)	64.5 (3.9)	66.4 (4.6)	64.4 (3.5)	0.61
*Endpoint*	62.3 (4.2)	66.7 (3.8)	70.9 (4.5)	66.6 (2.6)	0.48
*Difference*	3.2 (3.52	2.2 (3.0)	4.5 (3.2)	2.2 (3.2)	0.95
No. (%) Iron deficiency using adjusted ferritin values					
*Baseline*	9 (15.2)	3 (5.4)	4 (7.1)	1 (1.7)	0.60
*Endpoint*	5 (8.6)	3 (5.4)	4 (7.1)	3 (5.1)	0.48
*Difference*	4 (6.6)	0 (0.0)	0 (0.0)	2 (3.4)	
Serum transferrin receptors (mg/L)					
*Baseline*	7.2 (0.3)	6.8 (0.2)	6.7 (0.2)	6.9 (0.26)	0.54
*Endpoint*	7.2 (0.3	7.0 (0.3)	6.9 (0.2)	7.0 (0.31)	0.83
*Difference*	0.0 (0.2)	0.2 (0.2)	0.2(0.1)	0.1 (0.1)	
Low serum transferrin receptor (*n* and %)					
*Baseline*	10 (17.2)	6 (10.7)	5 (8.9)	8 (13.8)	0.56
*Endpoint*	9 (15.5)	10 (17.9)	7 (12.5)	8 (13.8)	0.87
*Difference*	1 (1.7)	4 (7.1)	2 (3.6)	0 (0.0)	
Body iron stores (mg/kg/body weight)					
*Baseline*	6.0 (0.34)	6.6 (0.26)	6.7 (0.29)	6.59 (0.24)	0.338
*Endpoint*	6.0 (0.35)	6.7 (0.26)	6.8 (0.24)	6.8 (0.2)	0.128
*Difference*	0.0 (0.2)	0.06 (0.18)	0.09 (0.24)	0.21 (0.19)	

^1^ *f*-test using one-way ANOVA; * significant at α = 0.05; ^a^ Adjusted ferritin level was done using the correction factors by stage (Thurnham CF).

**Table 4:** Mean of secondary biomarkers of study children by group by period.

Biochemical	Control	Low Dose	Moderate Dose	High Dose	*p*-Value ^1^
	(*n* = 58)	(*n* = 56)	(*n* = 56)	(*n* = 58)	
	Mean (SE)	Mean (SE)	Mean (SE)	Mean (SE)	
*RBP-(µmol/L) ^a^*					
Baseline	0.94 (0.03)	1 (0.03)	1.03 (0.04)	1.00 (0.03)	0.16
Endpoint	1.05 (0.03)	1.11 (0.04)	1.11 (0.03)	1.11 (0.03)	0.48
Difference	0.12 (0.03)	0.11 (0.04)	0.07 (0.03)	0.10 (0.03)	
*Zinc-(µg/dL) ^b^*					
Baseline	64 (3)	63 (3)	64 (3)	64 (2)	0.98
Endpoint	68 (2)	66 (2)	73 (3)	67 (3)	0.24
Difference	4 (3)	3 (3)	9 (4)	3 (3)	
*Vitamin C-(μg/dL) ^c^*					
Baseline	0.22 (0.01)	0.23 (0.02)	0.24 (0.02)	0.22 (0.02)	0.74
Endpoint	0.24 (0.02)	0.28 (0.02)	0.36 (0.03)	0.30 (0.02)	0.01 *
Difference	0.02 (0.02)	0.05 (0.02)	0.11 (0.02)	0.08 (0.02)	
*p*-value ^2^	0.23	0.01	0.00	0.00	
*Vitamin D-(nmol/L) ^d^*					
Baseline	60 (2)	64 (2)	64 (3)	69 (3)	0.44
Endpoint	65 (2)	68 (2)	69 (2)	73 (2)	0.11
Difference	5 (1)	3 (2)	5 (2)	7 (1)	

* Significant at α = 0.05; ^1^ *f*-test using one-way ANOVA; ^2^ *t*-test using paired samples; ^a^ *p* < 0.7 µmol/L defines Low *RBP* and between 0.7 µmol/L and 1.05 µmol/L for moderate deficiency; ^b^ *p* < 65 µg/dL (9.9 μmol/L) defines zinc deficiency; ^c^ *p* < 0.20 µg/dL defines vit. C deficiency; ^d^ *p* < 75 nmol/L defines vit. D deficiency.

**Table 5:** Mean per capita nutrient intake of Children and Percentage meeting the EAR with fortified juice drink by group by period control.

Nutrients	Control	%	Low Dose	%	Moderate Dose	%	High Dose	%
	(*n* = 58)		(*n* = 56)		(*n* = 56)		(*n* = 58)	
	Mean ± SE		Mean ± SE		Mean ± SE		Mean ± SE	
Vitamin A (µg RE)								
*Baseline*	499.8 ± 131.1	51.7	470.5 ± 93.6	64.3	734.7 ± 338.3	58.9	404.1 ± 43.4	69
*Endpoint*	484.6 ± 86.7	62.1	370.7 ± 27.0	62.5	384.3 ± 33.6	55.4	360.1 ± 26.0	67.2
*Difference ^1^*	−15.2 ± 158.8	10.3	−99.8 ± 99.7	−1.8	−350.4 ± 344.5	−3.6	−44.0 ± 47.8	−1.7
*Percent Contribution of juice drink*		1.6		2.1		2.0		2.2
Vitamin C (mg)								
*Baseline*	14.8 ± 2.4	22.4	26.2 ± 6.5	32.1	26.1 ± 6.7	30.4	49.5 ± 30.3	36.2
*Endpoint*	17.1 ± 2.8	25.9	40.7 ± 3.6	80.4	58.5 ± 6.7	100	60.9 ± 2.3	100
*Difference ^1^*	2.4 ± 3.3	3.4	14.5 ± 7.6	48.2 *	32.4 ± 9.4 *	69.6 *	11.4 ± 30.6	63.8 *
*Percent Contribution of juice drink*		16.8		68.6		74.0		87.6
Thiamin (mg)								
*Baseline*	0.7 ± 0.1	48.3	0.9 ± 0.1	62.5	0.8 ± 0.07	60.7	0.9 ± 0.1	74.1
*Endpoint*	0.8 ± 0.1	55.2	0.8 ± 0.1	66.1	1.0 ± 0.1	76.8	1.1 ± 0.1	82.8
*Difference ^1^*	0.1 ± 0.1	6.9	0.1 ± 0.1	3.6	0.2 ± 0.1 *	16.1 *	0.2 ± 0.1	8.6
*Percent Contribution of juice drink*		1.3		20.0		25.7		32.8
Riboflavin (mg)								
*Baseline*	0.6 ± 0.1	39.7	0.8 ± 0.07	57.1	0.8 ± 0.1	58.9	0.8 ± 0.1	63.8
*Endpoint*	0.7 ± 0.1	55.2	0.7 ± 0.04	69.6	0.9 ± 0.1	78.6	0.8 ± 0.0	81
*Difference ^1^*	0.1 ± 0.1	15.5 *	−0.0 ± 0.1	12.5	0.0 ± 0.1	19.6 *	0.0 ± 0.1	17.2 *
*Percent Contribution of juice drink*		0.0		18.5		24.6		33.3
Niacin (mg)								
Baseline*	10.2 ± 0.6	70.7	12.5 ± 1.18	73.2	12.0 ± 1.2	76.8	14.6 ± 1.2	87.9
Endpoint*	11.2 ± 0.8	69	13.0 ± 0.64	94.6	13.7 ± 0.2	96.4	16.0 ± 0.8	98.3
*Difference*	1.0 ± 1.0	−1.7	0.5 ± 1.38	21.4 *	1.7 ± 1.3	19.6 *	1.4 ± 1.3	10.3 *
*Percent Contribution of juice drink*		0.2		20.8		30.9		35.9
Calcium (mg)								
*Baseline*	284.3 ± 27.4	13.8	314.4 ± 23.3	19.6	373.6 ± 35.2	35.7	332.3 ± 24.8	19
*Endpoint*	295.3 ± 26.8	15.5	342.1 ± 22.3	19.6	387.8 ± 33.2	30.4	359.1 ± 18.1	25.9
*Difference ^1^*	11.0 ± 27.4	1.7	27.6 ± 26.2	0	14.3 ± 36.8	−5.4	26.7 ± 28.3	6.9
*Percent Contribution of juice drink*		0.0		17.6		24.8		35.3
Iron (mg)								
*Baseline*	7.0 ± 0.4	29.3	7.7 ± 0.57	39.3	8.4 ± 0.9	35.7	7.8 ± 0.5	36.2
*Endpoint*	7.6 ± 0.6	29.3	9.0 ± 0.49	53.6	9.1 ± 0.5	48.2	10.1 ± 0.5	62.1
*Difference ^1^*	0.6 ± 0.8	0	1.2 ± 0.8	14.3	0.6 ± 1.1	12.5	2.4 ± 0.7 *	25.9 *
*Percent Contribution of juice drink*		0.3		23.1		35.4		42.3

^1^ *f*-test using one-way ANOVA; * significant at α = 0.05.
